# Dietary Recommendations for the Prevention of Type 2 diabetes: What Are They Based on?

**DOI:** 10.1155/2012/847202

**Published:** 2012-01-19

**Authors:** Patrice Carter, Kamlesh Khunti, Melanie J. Davies

**Affiliations:** ^1^Diabetes Research, Department of Cardiovascular Sciences, University of Leicester, Leicester LE1 5WW, UK; ^2^Department of Health Sciences, University of Leicester, Leicester LE1 5WW, UK

## Abstract

*Introduction*. Type 2 diabetes is increasing in all populations and all age groups across the world. Areas undergoing rapid westernisation and rapid nutrition transition are seeing the greatest increases in prevalence suggesting that environmental factors are important. Studies from around the world have shown that dietary modification for the prevention of T2DM can be successful; however which dietary factors are important remains to be fully elucidated. The WHO, ADA, and Diabetes UK have developed guidelines for the prevention of T2DM even though the WHO states that data from lifestyle modification programmes does not allow for the disentanglement of dietary factors. *Aim of Review*. The aim of this focused review is to evaluate the current dietary recommendations for the prevention of T2DM. In addition we aim to explore the available evidence from both observation studies and clinical trials to determine whether these recommendations are appropriate.

## 1. Introduction

The prevalence of Type 2 diabetes (T2DM) is currently estimated to be 6.4% worldwide [1] and is increasing in all populations and all age groups throughout the world. This rapid increase in prevalence over the last two decades suggests that environmental factors must be important. In addition populations undergoing rapid lifestyle changes and rapid nutrition transition have seen the greatest increase in prevalence [2]. Since 1921 primary prevention of T2DM has been implicated as an important way to tackle T2DM [3], and over recent years a number of trials have provided evidence to support the theory that T2DM is a preventable and potentially reversible disease [4–9]. Indeed there is now overwhelming evidence to conclude that lifestyle modification can prevent or delay the onset of T2DM [10]. In addition it has also been demonstrated that diet alone can significantly reduce the incidence of T2DM [10]. It is currently unclear which dietary changes are important; yet the ADA, WHO, and Diabetes UK have set dietary guidelines for the prevention of T2DM [11–13]. Both the Finnish Diabetes Prevention study and the Diabetes Prevention Programme reported a decrease in average fat intake in individuals enrolled into treatment arms [6, 14] and the FDPS also reported an increase in percentage energy from carbohydrates and fibre, resulting in the authors to conclude that their findings support current dietary recommendations [14]. However the WHO states that the data from these lifestyle modification programmes (LSMP) does not allow for the disentanglement of dietary aspects to determine which nutrients are important for the prevention of T2DM [11]. Therefore the question arises, where do the dietary recommendations for the prevention of T2DM come from and what is the evidence behind them?

## 2. Current WHO and ADA Recommendations

It is widely accepted that obesity is the single most important risk factor for T2DM; being overweight, having abdominal fat distribution, and obesity account for around 90% of all T2DM cases [15]. Guidelines emphasise the need to maintain a healthy weight (BMI <25 kg/m^2^) or for those overweight to reduce weight to prevent T2DM [11–13]. However physical activity has also been identified as an important risk factor in the development of T2DM independently of obesity [16].

The ADA suggests that those developing lifestyle modification programmes for people at risk of T2DM should recommend reducing dietary fat and increasing physical activity to reduce weight. The only direct recommendation for dietary changes is that those at risk of T2DM should achieve 14 g per 1000 kcal of fibre intake and that whole grains make up half of total grain intake [12]. However, the WHO only states that there is probable evidence that intake of nonstarch polysaccharides reduces risk. The WHO claims that the only convincing evidence for the dietary prevention of T2DM is weight loss [11].

The advice for prevention of T2DM is in fact very similar to that given to the general population for a healthy diet [17]. The Network to Prevent Diabetes Group (IMAGE project) have developed a toolkit which provides dietary advice for prevention of T2DM [18], which is nevertheless similar to that for the general population. So is there evidence that those at high risk of T2DM should eat anything different to prevent or delay the onset of T2DM?

## 3. Dietary Fibre

In regards to dietary fibre, as recommended by the ADA [12], the evidence from epidemiological studies is fairly compelling. Both the Nurses Health Study and the Iowa Womens Health study found strong inverse relationships between whole grain intake and T2DM incidence, observing 38% and 21% reduced incidence with increased consumption [19, 20]. The Male Health Professionals followup study also found an inverse relationship between whole grain intake and incidence of T2DM; however the relationship was largely explained by cereal fibre [21]. A recent report examined both the Nurses Health study I and II plus the Male Health Professionals followup study and specifically investigated the potential role that white and brown rice may have on the development of T2DM [22]. Brown rice being a rich source of whole grains as compared to white rice, which has the majority of the grain removed. The data showed that greater consumption of white rice was associated with an increased risk of T2DM while greater intake of brown rice was associated with reduced risk of developing T2DM. Substituting brown rice for white rice decreased risk of T2DM independently of other lifestyle factors, supporting the role of whole grains for the prevention of T2DM [22]. A meta-analysis of prospective studies examining whole grains and incidence T2DM also reported that studies consistently found whole grain intake to reduce the incidence of T2DM. Pooled summary estimates from six epidemiological studies showed that for each two serving increase per day there was a 21% reduction in risk of T2DM (RR 0.79, 95% CI 0.72–0.87) [23].

When determining relative risks between consumption of whole grains and T2DM adjustment for BMI and physical activity must be considered. The meta-analysis carried out by de Munter et al. found adjustment for BMI substantially weakened observed associations between whole grain intake and T2DM [23]. Doubtlessly intake of dietary fibre is also strongly linked to BMI, with whole grain consumption protecting against weight gain [24]. Fung et al. demonstrated differences in effects of whole grains depending on an individual's weight status. In obese subjects whole grain consumption was only weakly associated to the incidence of T2DM but in those with a BMI < 30 kgm^2^ a higher intake resulted in 50% reduced incidence when comparing highest to lowest consumption [21].

Studies also exist which demonstrate that a change in whole grain consumption can influence glucose and insulin levels. A crossover study provided obese subjects with two six-week feeding periods and reported significantly lower fasting insulin levels during the whole grain feeding period compared to the refined grain feeding period. There was also a tendency for fasting glucose to be lower in the whole grain period but the results were not significant [25]. Aller et al. suggest that modest increases in soluble fibre can affect glucose levels; subjects without T2DM increased fibre in the diet which resulted in a 12.3% reduction in glucose levels [26]. However fibre intake was increased via an increase in breakfast cereal and by consuming 2 apples per day; the study claims that fibre was the influencing factor; yet it cannot exclude other potential benefits of increased apple consumption. It is well documented that fruits are a rich source of antioxidants which may play a role in glucose control [27]. A more robustly designed study enriched different breakfast meals with oat powder, rye bran, or sugar beet fibre; the fat and carbohydrate content of the meals was balanced by rapeseed oil and dextrose powder. All test meals reduced incremental postprandial glucose compared to the control meal; however only the rye bran meal showed significant improvements [28].

There are a number of potential mechanisms by which dietary fibre may play a role within insulin sensitivity and glucose tolerance. Fibre can impact on intestinal tract time, absorption of macronutrients, alter the action of digestive enzymes and secretion of gastrointestinal and pancreatic hormones [29]. Insoluble fibre can decrease intestinal tract time, potentially reducing time for the carbohydrates to be absorbed in the jejunum [30]. Soluble fibre delays gastric emptying slowing the absorption and digestion of carbohydrates potentially delaying the insulin response [30]. In addition non digestive fibre is fermented by the microflora of the colon resulting in the production of short chain fatty acids which may also impact on carbohydrate metabolism [31].

Although the data on whole grain intake and risk of T2DM appears consistent, a Cochrane review concluded that as most evidence is from observational studies where confounding factors cannot be ruled out, more research is needed before whole grains can truly be termed preventative [32]. In addition the Scientific Advisory Committee on Nutrition states that studies are inconsistent and inconclusive; so firm recommendations cannot be made regarding fibre and whole grains and their association with T2DM [33]. Therefore it seems that more research into whole grain consumption is required before their role in prevention of T2DM will be accepted by governing bodies. Studies need to be devised that independently increase intake of whole grains in those with low consumption levels.

## 4. Dietary Fat

Reduction of saturated fatty acid (SFA) intake is generally included in all dietary recommendations to prevent T2DM; however evidence is contradictory and inconclusive. Plasma membranes are composed of lipids and membrane properties are not fixed but highly adaptive [34]; therefore incorporation of different fat types into cell membranes can potentially alter the membrane's physical properties and function. Changing the phospholipid bilayer can affect membrane fluidity and cellular functions and influence carrier-mediated receptors and the properties of membrane bound enzymes [35, 36]. Insulin receptors are embedded in the lipid bilayer of plasma membranes of cells, including both skeletal and adipose cells [37]; thus altering dietary fat may influence insulin sensitivity. Indeed rodent studies have supported this theory; rats fed high fat diets go onto develop insulin resistance in both skeletal and adipose cells [38, 39]. In addition a number of human studies add to the evidence. A study in men with normal glucose tolerance demonstrated that arachidonic acid was positively correlated to 2-hour glucose while SFAs were negatively correlated to insulin secretion [35]. A study in older men also showed FA composition of both serum and skeletal muscle to be affected by the level of SFA, with a strong negative relationship between palmitic acid and content of skeletal muscle lipids and insulin sensitivity [40]. The San Luis study demonstrated that those with impaired glucose tolerance who consumed greater amounts of total fat had an increased risk of developing T2DM independent of obesity, insulin, and glucose levels at baseline [41]. Further supporting the role of fat type in the diet is the KANWU study, a randomized controlled trial where participants were randomized to receive diets high in SFA or Monounsaturated fatty acids (MUFAs) [42]. Changes in serum phospholipids reflected the two test diets and insulin sensitivity was significantly decreased in the group consuming high SFA diet. However insulin sensitivity was unchanged in the MUFA group. The study also randomly allocated placebo tables of n-3 fish oils to participants; however addition of n-3 to the diet had no effect on insulin sensitivity [42].

These studies support the biological plausibility that dietary fat can influence insulin sensitivity; however a number of large epidemiological studies have been less conclusive. The Nurses Health Study found only weak associations between total fat, SFA, and the risk of developing T2DM; however polyunsaturated fatty acids (PUFAs) were inversely related to incidence of T2DM [43]. Similar results were observed in the Iowa Womens Health study [44]. The strongest relationship observed in the Nurses Health study was between trans fatty acid consumption and incidence T2DM, with each 2% increase in trans fat increasing the risk of T2DM by 31% (RR 1.31 95% CI 1.10–1.56) [43]. In addition the Male Health Professional followup study found a positive relationship between total fat, SFA, and incidence of T2DM. However relationships were attenuated by adjustment for cereal fibre and lost once BMI was incorporated into the model [45]. The close association between fat intake and obesity and between obesity and T2DM may make it difficult to disentangle small differences. The majority of observational studies rely on food frequency questionnaires (FFQ) to determine dietary intake; however measurement errors can underestimate true interactions between diet and disease [46, 47]. It has previously been demonstrated that FFQs were unable to detect relationships between fat intake and breast cancer while other assessment methods have shown associations [48]. In addition self-report of fat intake may also be open to criticism as it has been well documented that overweight individuals underreport intake [49].

The true relationship between fat type, insulin resistance, and T2DM remains to be fully elucidated and further exploration is needed. Further trials such as the KANWU and San Luis study should be developed to enable true assessment of fat intake and risk of T2DM. It will also be important to carry these investigations out in both lean and obese individuals.

## 5. Other Dietary Factors

Lifestyle modification programs carried out to prevent T2DM have not simply increased fibre intake and reduced fat; they have also included recommendations to increase fruit and vegetable intake [4, 6, 7]. A review recently unequivocally found green leafy vegetables to be protective from the development of T2DM, with all studies included found to have a protective effect from increased intake [50]. Results for other fruit and vegetables were less conclusive; however the authors claimed that studies were generally of low quality and heterogeneous. Again the reliance on FFQ may have potentially attenuated true relationships [50].

There is growing evidence that processed meat may be detrimental to health and greater consumption may increase the risk of T2DM. A meta-analysis of seven studies observed a 19% higher risk of T2DM with each serving of processed meat consumed per day [51]. A further study also found a positive association between processed meat intake and risk of T2DM (RR 1.41 95% CI 1.25–1.60) comparing highest to lowest intake [52].

## 6. Dietary Patterns

Determination of interactions between individual foods and disease is difficult to detect. In addition foods are rarely eaten in isolation but in combination with other food groups [53]. Indeed the combination of food groups may be interactive, creating an additive effect on health [54]. Analysis of dietary patterns rather than of individual food groups takes this food synergy into account [53]. Logically a number of studies have examined dietary patterns and the incidence of T2DM and results are largely consistent. Factor analysis was used to determine dietary patterns in the Male Health Professionals followup study [55]. Two patterns were identified, the prudent pattern based on high intakes of vegetables, legumes, fruit, whole grains, fish, and poultry and a Western pattern, characterized by red meat, processed meat, refined grains, French fries, high-fat diary, sweets, desserts, high-sugar drinks, and eggs. The prudent diet was only moderately associated with a reduced risk of T2DM but the Western pattern strongly increased risk of T2DM (RR 1.59, 95% CI 1.32–1.93) [55]. The prudent pattern was also shown to predict lower T2DM incidence over 23 years of followup in the Finnish Mobile Clinic Health Examination Survey [56]. Evidence appears to suggest that diets rich in fruit and vegetables and whole grains yet with a low intake of red meat, and SFA have reduced risk of T2DM [55–57]. The Mediterranean diet is one such diet to consist of these food types, high adherence to a Mediterranean diet in the SUN study was associated with an 83% reduced risk of T2DM, and this was despite a high prevalence of risk factors in these individuals [58]. The value of examining dietary patterns rather than individual food items is supported by the analysis of dietary intake in the Multiethnic Study of Atherosclerosis [59]. A dietary pattern characterized by high intake of whole grains, fruit, nuts, and seeds and green leafy vegetables and low fat dairy products was found to reduce diabetes risk by 15%; however no individual food group was independently associated with T2DM [59].

## 7. Conclusion

The current dietary recommendations for the prevention of T2DM are similar to those given to the general population for a healthy diet. The recommendations are appropriate to the evidence available. Although data on individual dietary items remains inconclusive, the analysis of dietary patterns is compelling. Studies consistently demonstrate that diets rich in fruit and vegetables, whole grains, legumes, and low in red and processed meat, SFA, and refined grains protect from T2DM development. Individuals who consume a healthy diet are also often more active; further research into the role of physical activity for the prevention of T2DM and its relationship with dietary habits should be encouraged.

Nutritional evidence largely originates from observational studies which are open to criticism due to confounding factors that cannot always be accounted for. In addition most observational studies rely on FFQ which are recognized as being insensitive to small yet significant interactions between diet and disease. Research into the nutrients contained within foods is also necessary. Magnesium intake has been identified as important for reducing T2DM incidence [60]. Magnesium is present in whole grains and vegetables, as is zinc another potentially important element involved in the development of insulin resistance and glucose tolerance; however clinical research is lacking.

Despite the plethora of studies, little new data on the role of nutrition and the prevention of T2DM has been produced over the past decade. Further robust randomized trials with larger samples sizes are needed. Trials which provide realistic intakes of food groups are needed to help elucidate true effect of diet on health. The additional use of biomarkers rather than reliance on FFQ is also warranted. Studies investigating which foods and nutrients influence the development of T2DM including studies which determine the mechanistic actions of foods and nutrients are needed before many recognized organizations will truly accept that the foods we consume can be preventative for T2DM.

## References

[1] Shaw JE, Sicree RA, Zimmet PZ (2010). Global estimates of the prevalence of diabetes for 2010 and 2030. *Diabetes Research and Clinical Practice*.

[2] Alberti KGMM, Zimmet P, Shaw J (2007). International Diabetes Federation: a consensus on Type 2 diabetes prevention. *Diabetic Medicine*.

[3] Lindström J, Uusitupa M (2008). Lifestyle intervention, diabetes, and cardiovascular disease. *The Lancet*.

[4] Pan XR, Li GW, Hu YH (1997). Effects of diet and exercise in preventing NIDDM in people with impaired glucose tolerance: the Da Qing IGT and diabetes study. *Diabetes Care*.

[5] Tuomilehto J, Lindström J, Eriksson JG (2001). Prevention of type 2 diabetes mellitus by changes in lifestyle among subjects with impaired glucose tolerance. *The New England Journal of Medicine*.

[6] Knowler WC, Barrett-Connor E, Fowler SE (2002). Reduction in the incidence of type 2 diabetes with lifestyle intervention or metformin. *The New England Journal of Medicine*.

[7] Ramachandran A, Snehalatha C, Mary S, Mukesh B, Bhaskar AD, Vijay V (2006). The Indian Diabetes Prevention Programme shows that lifestyle modification and metformin prevent type 2 diabetes in Asian Indian subjects with impaired glucose tolerance (IDPP-1). *Diabetologia*.

[8] Buchwald H, Avidor Y, Braunwald E (2004). Bariatric surgery: a systematic review and meta-analysis. *JAMA*.

[9] Lim EL, Hollingsworth KG, Aribisala BS, Chen MJ, Mathers JC, Taylor R (2011). Reversal of type 2 diabetes: normalisation of beta cell function in association with decreased pancreas and liver triacylglycerol. *Diabetologia*.

[10] Gillies CL, Abrams KR, Lambert PC (2007). Pharmacological and lifestyle interventions to prevent or delay type 2 diabetes in people with impaired glucose tolerance: systematic review and meta-analysis. *British Medical Journal*.

[11] http://www.who.int/dietphysicalactivity/publications/trs916/en/.

[12] Bantle JP, Wylie-Rosett J, Albright AL (2008). Nutrition recommendations and interventions for diabetes. A position statement of the american diabetes association. *Diabetes Care*.

[13] Dyson PA, Kelly T, Deakin T (2011). Diabetes UK evidence-based nutrition guidelines for the prevention and management of diabetes. *Diabetic Medicine*.

[14] Lindström J, Louheranta A, Mannelin M (2003). The Finnish Diabetes Prevention Study (DPS): lifestyle intervention and 3-year results on diet and physical activity. *Diabetes Care*.

[15] Astrup A, Finer N (2000). Redefining type 2 diabetes: “Diabesity” or “obesity dependent diabetes mellitus”?. *Obesity Reviews*.

[16] Laaksonen MA, Knekt P, Rissanen H (2010). The relative importance of modifiable potential risk factors of type 2 diabetes: a meta-analysis of two cohorts. *European Journal of Epidemiology*.

[17] http://www.nhs.uk/Livewell/Goodfood/Pages/eatwell-plate.aspx.

[18] http://www.idf.org/webdata/docs/idf-europe/IMAGE%20Toolkit.pdf.

[19] Liu S, Manson JE, Stamfer MJ (2000). A prospective study of whole-grain intake and risk of type 2 diabetes mellitus in US women. *American Journal of Public Health*.

[20] Meyer KA, Kushi LH, Jacobs DR, Slavin J, Sellers TA, Folsom AR (2000). Carbohydrates, dietary fiber, and incident type 2 diabetes in older women. *American Journal of Clinical Nutrition*.

[21] Fung TT, Hu FB, Pereira MA (2002). Whole-grain intake and the risk of type 2 diabetes: a prospective study in men. *American Journal of Clinical Nutrition*.

[22] Sun Q, Spiegelman D, Van Dam RM (2010). White rice, brown rice, and risk of type 2 diabetes in US men and women. *Archives of Internal Medicine*.

[23] de Munter JSL, Hu FB, Spiegelman D, Franz M, Van Dam RM (2007). Whole grain, bran, and germ intake and risk of type 2 diabetes: a prospective cohort study and systematic review. *PLoS Medicine*.

[24] Koh-Banerjee P, Franz M, Sampson L (2004). Changes in whole-grain, bran, and cereal fiber consumption in relation to 8-y weight gain among men. *American Journal of Clinical Nutrition*.

[25] Pereira MA, Jacobs DR, Pins JJ (2002). Effect of whole grains on insulin sensitivity in overweight hyperinsulinemic adults. *American Journal of Clinical Nutrition*.

[26] Aller R, de Luis DA, Izaola O (2004). Effect of soluble fiber intake in lipid and glucose leves in healthy subjects: a randomized clinical trial. *Diabetes Research and Clinical Practice*.

[27] Giugliano D, Esposito K (2008). Mediterranean diet and metabolic diseases. *Current Opinion in Lipidology*.

[28] Ulmius M, Johansson A, Önning G (2009). The influence of dietary fibre source and gender on the postprandial glucose and lipid response in healthy subjects. *European Journal of Nutrition*.

[29] Anderson JW (1986). Fiber and health: an overview. *American Journal of Gastroenterology*.

[30] Montonen J, Knekt P, Järvinen R, Aromaa A, Reunanen A (2003). Whole-grain and fiber intake and the incidence of type 2 diabetes. *American Journal of Clinical Nutrition*.

[31] Thorburn A, Muir J, Proietto J (1993). Carbohydrate fermentation decreases hepatic glucose output in healthy subjects. *Metabolism*.

[32] Priebe MG, van Binsbergen JJ, de Vos R, Vonk RJ (2008). *Whole Grain Foods for the Prevention of Type 2 Diabetes Mellitus (Review)*.

[33] http://www.sacn.gov.uk/pdfs/final_sacn_position_statement_for_website_dietary_fibre.pdf.

[34] Hulbert AJ, Turner N, Storlien LH, Else PL (2005). Dietary fats and membrane function: implications for metabolism and disease. *Biological Reviews of the Cambridge Philosophical Society*.

[35] Pelikanova T, Kohout M, Valek J, Base J, Kazdova L (1989). Insulin secretion and insulin action related to the serum phospholid fatty acid pattern in healthy men. *Metabolism*.

[36] Haag M, Dippenaar NG (2005). Dietary fats, fatty acids and insulin resistance: short review of a multifaceted connection. *Medical Science Monitor*.

[37] Field CJ, Ryan EA, Thomson ABR, Clandinin MT (1988). Dietary fat and the diabetic state alter insulin binding and the fatty acyl composition of the adipocyte plasma membrane. *Biochemical Journal*.

[38] Storlien LH, Baur LA, Kriketos AD (1996). Dietary fats and insulin action. *Diabetologia*.

[39] Fickova M, Hubert P, Crémel G, Leray C (1998). Dietary (n-3) and (n-6) polyunsaturated fatty acids rapidly modify fatty acid composition and insulin effects in rat adipocytes. *Journal of Nutrition*.

[40] Vessby B, Tengblad S, Lithell H (1994). Insulin sensitivity is related to the fatty acid composition of serum lipids and skeletal muscle phospholipids in 70-year-old men. *Diabetologia*.

[41] Marshall JA, Hoag S, Shetterly S, Hamman RF (1994). Dietary fat predicts conversion from impaired glucose tolerance to NIDDM: the San Luis Valley Diabetes Study. *Diabetes Care*.

[42] Vessby B, Uusitupa M, Hermansen K (2001). Substituting dietary saturated for monounsaturated fat impairs insulin sensitivity in healthy men and women: the KANWU study. *Diabetologia*.

[43] Salmerón J, Hu FB, Manson JE (2001). Dietary fat intake and risk of type 2 diabetes in women. *American Journal of Clinical Nutrition*.

[44] Meyer KA, Kushi LH, Jacobs DR, Folsom AR (2001). Dietary fat and incidence of type 2 diabetes in older Iowa women. *Diabetes Care*.

[45] Van Dam RM, Willett WC, Rimm EB, Stampfer MJ, Hu FB (2002). Dietary fat and meat intake in relation to risk of type 2 diabetes in men. *Diabetes Care*.

[46] Kipnis V, Subar AF, Midthune D (2003). Structure of dietary measurement error: results of the OPEN biomarker study. *American Journal of Epidemiology*.

[47] Prentice RL (2003). Dietary assessment and the reliability of nutritional epidemiology reports. *The Lancet*.

[48] Bingham SA, Luben R, Welch A, Wareham N, Khaw KT, Day N (2003). Are imprecise methods obscuring a relation between fat and breast cancer?. *The Lancet*.

[49] Weber JL, Reid PM, Greaves KA (2001). Validity of self-reported energy intake in lean and obese young women, using two nutrient databases, compared with total energy expenditure assessed by doubly labeled water. *European Journal of Clinical Nutrition*.

[50] Carter P, Gray LJ, Troughton J, Khunti K, Davies MJ (2010). Fruit and vegetable intake and incidence of type 2 diabetes mellitus: systematic review and meta-analysis. *BMJ*.

[51] Micha R, Wallace SK, Mozaffarian D (2010). Red and processed meat consumption and risk of incident coronary heart disease, stroke, and diabetes mellitus: a systematic review and meta-analysis. *Circulation*.

[52] Aune D, Ursin G, Veierød MB (2009). Meat consumption and the risk of type 2 diabetes: a systematic review and meta-analysis of cohort studies. *Diabetologia*.

[53] Jinlin F, Binyou W, Terry C (2007). A new approach to the study of diet and risk of type 2 diabetes. *Journal of Postgraduate Medicine*.

[54] Jacobs DR, Steffen LM (2003). Nutrients, foods, and dietary patterns as exposures in research: a framework for food synergy. *American Journal of Clinical Nutrition*.

[55] Van Dam RM, Rimm EB, Willett WC, Stampfer MJ, Hu FB (2002). Dietary patterns and risk for type 2 diabetes mellitus in U.S. men. *Annals of Internal Medicine*.

[56] Montonen J, Knekt P, Härkänen T (2005). Dietary patterns and the incidence of type 2 diabetes. *American Journal of Epidemiology*.

[57] Hodge AM, English DR, O’Dea K, Giles GG (2007). Dietary patterns and diabetes incidence in the Melbourne collaborative cohort study. *American Journal of Epidemiology*.

[58] Martínez-González MA, De La Fuente-Arrillaga C, Nunez-Cordoba JM (2008). Adherence to Mediterranean diet and risk of developing diabetes: prospective cohort study. *BMJ*.

[59] Nettleton JA, Steffen LM, Ni H, Liu K, Jacobs DR (2008). Dietary patterns and risk of incident type 2 diabetes in the multi-ethnic study of atherosclerosis (MESA). *Diabetes Care*.

[60] Larsson SC, Wolk A (2007). Magnesium intake and risk of type 2 diabetes: a meta-analysis. *Journal of Internal Medicine*.

